# Book Reviews

**Published:** 1903-09

**Authors:** 


### BOOK REVIEWS.

GYNECOLOGY. A Text-book for Students, and a Guide for Practitioners. By Wm. R. Pryor, M.D., New York. One hundred and sixty-three illustrations in the text. D. Appleton & Co., New York and London, 1903.

This book deals with diseases peculiar to women, their operative and non-operative treatment. The author has striven to make the work of a practical character, omitting all that is not strictly confined to the domain of gynecology and within this domain employing only such subject-matter as strictly pertains to it. The book is consequently brief, but much to the point. The teaching is clear and convincing and the operative descriptions admirably lucid, being further aided by numerous fine illustrations. The book can be heartily commended for students, and also for more advanced workers in this field.

---

THE AMERICAN CONGRESS ON TUBERCULOSIS FOR THE PREVENTION OF CONSUMPTION.

SECRETARY'S OFFICE,
ATLANTA, GA., U. S. A., August 1, 1903.

*To the Members of the Medical Profession :*

The President of the American Congress on Tuberculosis, to be held in Washington, D. C., April 4th, 5th and 6th, 1905, announces Dr. Alfred Meyer, of New York City, consulting physician to the Bedford Sanitarium for Consumptives, Chairman of a committee in charge of the section on Sanitarium treatment of tuberculosis. It is probable that the climatic, and other methods of treatment will be comprised under the work of this committee.

Very truly yours,

GEORGE BROWN, M.D.,
Secretary American Congress on Tuberculosis.

